# Hyperinsulinemic hypoglycemia due to pathogenic INSR variants: metabolic signature, phenotypic overlap, and semidominant inheritance

**DOI:** 10.3389/fendo.2026.1716020

**Published:** 2026-05-07

**Authors:** Ramon Marcelino do Nascimento, Andrey dos Santos, Dioze Guadagnini, Lucas Santos de Santana, Augusto Cezar Junior Santomauro, Caroline Gouveia Buff Passone, Milena Gurgel Teles Bezerra, Larissa Garcia Gomes, Maria Lucia Corrêa-Giannella, Marcia Nery, Mario José Abdalla Saad, Delmar Muniz Junior Lourenço, Maria Adelaide Albergaria Pereira

**Affiliations:** 1 Division of Endocrinology and Metabolism, University of São Paulo School of Medicine (FMUSP), São Paulo, Brazil; 2 Department of Internal Medicine, School of Medical Science, State University of Campinas (UNICAMP), Campinas, SP, Brazil; 3 Endocrine Genetics Unit, Laboratory of Cellular and Molecular Endocrinology (LIM-25), Hospital das Clínicas, University of São Paulo School of Medicine (FMUSP), São Paulo, Brazil; 4 Instituto da Criança e do Adolescente, University of São Paulo - Pediatric Endocrinology Department, São Paulo, Brazil; 5 Laboratory of Hormones and Molecular Genetics (LIM-42), Department of Internal Medicine, Division of Endocrinology and Metabolism, Adrenal Unit, Hospital das Clínicas, University of São Paulo School of Medicine (FMUSP), São Paulo, Brazil; 6 Laboratory of Carbohydrates and Radioimmunoassay (LIM-18), Hospital das Clínicas, University of São Paulo School of Medicine (FMUSP), São Paulo, Brazil

**Keywords:** exoma, familial hyperinsulinemic hypoglycemia, *INSR*, ketonemia, type A insulin resistance

## Abstract

**Objective:**

Familial hyperinsulinemic hypoglycemia type 5 (HHF5) and type A insulin resistance syndrome (TAIRS) are autosomal dominant disorders caused by heterozygous pathogenic variants in the insulin receptor gene (*INSR*), whereas Rabson–Mendenhall syndrome (RMS) and Donohue syndrome (DS) result from recessive inheritance. We aimed to characterize the metabolic, phenotypic, and genotypic spectrum of individuals with PGV-*INSR* presenting with hyperinsulinemic hypoglycemia (HH) and insulin resistance (IR).

**Methods:**

Normal-weight probands with HH and IR underwent exome sequencing with familial segregation confirmed by Sanger sequencing. Metabolic evaluation included prolonged fasting, mixed-meal, and oral glucose tolerance tests. A published insulinoma cohort served as a comparator.

**Results:**

Five unrelated families (F1–F5) harbored four heterozygous and one homozygous PGV-*INSR*. Eighteen carriers were identified. HH occurred in 78% (18/23), predominantly postprandial, whereas 22% had diabetes; IR was present in all carriers and 60% of women had PCOS. Marked intrafamilial variability and phenotypic overlap among HHF5, TAIRS, and RMS were observed, including the coexistence of dominant and recessive inheritance within one family. A distinct metabolic signature was identified, characterized by normal BMI, variable degrees of IR (with or without acanthosis), inappropriately elevated fasting and stimulated insulin concentrations, normal triglycerides, and predominantly postprandial hypoglycemia paradoxically associated with ketonemia.

**Conclusion:**

PGV-*INSR* defines a paradoxical metabolic phenotype combining hypoglycemia with ketonemia and insulin resistance, accompanied by broad intrafamilial variability. Recognition of this pattern may support targeted *INSR* testing, refine genetic counseling, and improve clinical management.

## Introduction

Hyperinsulinemic hypoglycemia (HH) is a rare condition with heterogeneous etiologies. Its estimated incidence is approximately 1 in 28,000 individuals (1). The most common causes include sporadic and inherited insulinomas, non-insulinoma pancreatogenous hypoglycemia syndrome (NIPHS), autoimmune hypoglycemia due to anti-insulin or anti-insulin receptor antibodies, factitious hypoglycemia induced by exogenous insulin or secretagogues, and post-bariatric surgery hypoglycemia (2–15). More rarely, HH has been associated with insulinomatosis, a distinct entity characterized by multiple insulin-secreting tumors (16). An even more exceptional etiology involves pathogenic germline variants (PGVs) in the *INSR* gene, one of 19 exceedingly rare genes known to cause HH (17).

A broad clinical spectrum of *INSR*-related phenotypes has been described, classically encompassing type A insulin resistance syndrome (TAIRS), familial hyperinsulinemic hypoglycemia type 5 (HHF5), Rabson–Mendenhall syndrome (RMS), and Donohue syndrome (DS) (3, 18–20). Inactivating *INSR* pathogenic germline variants (PGV-*INSR*) are typically associated with insulin resistance (IR), glucose intolerance, or diabetes, but in rare instances may manifest predominantly with fasting or postprandial hypoglycemia, as observed in HHF5. Both TAIRS and HHF5 result from monoallelic *INSR* variants and follow autosomal dominant inheritance, whereas the syndromic forms RMS and DS arise from biallelic variants and follow autosomal recessive inheritance.

More recently, Collin-Chavagnac et al. (2025) (21), observing marked phenotypic variability among five heterozygous parents of patients with RMS, proposed that *INSR*-related disorders may follow a semidominant model of inheritance. In this model, heterozygous carriers (with one PGV-*INSR* and one wild-type allele) exhibit an intermediate or milder phenotype compared with homozygous or compound heterozygous individuals, who develop more severe disease manifestations.

Since its initial description in a three-generation Danish family comprising 10 affected members, only a few additional families with HHF5 have been reported worldwide, mostly as isolated case reports. HH is the hallmark feature of HHF5 and often the first clinical manifestation, typically accompanied by IR features such as acanthosis nigricans in individuals with a normal body mass index (BMI) (22). Conversely, HH appears to be uncommon in patients with TAIRS, DS, or RMS.

The classical hyperinsulinemic hypoglycemia profile is characterized by inappropriate insulin secretion that persists despite low plasma glucose concentrations, reflecting a dissociation between glucose metabolism and insulin release by pancreatic β cells, with suppressed ketone bodies consistent with the anabolic effects of hyperinsulinemia. This biochemical profile is shared by the classical familial forms of hyperinsulinemic hypoglycemia caused by pathogenic variants in genes such as *ABCC8*, *KCNJ11*, *GLUD1*, *GCK*, *HADH*, *SLC16A1*, *UCP2*, *HNF4A*, *HNF1A*, *HK1*, *PGM1*, *PPM2, CACNA1D*, and *FOXA2*, in which the primary defect lies in dysregulated insulin secretion at the level of β cells (23).

Despite these observations, systematic studies addressing the occurrence and glico-insulinemic and ketotic profiles of hypoglycemia across *INSR*-related phenotypes remain scarce. Here, we report 18 additional cases from five families carrying molecularly confirmed PGV-*INSR*, representing the largest single-center series to date. Among the five variants identified, two deserve special mention: one previously unreported and another previously linked only to polycystic ovary syndrome (PCOS). This approach enabled a detailed clinical and metabolic characterization of affected individuals, complemented by comparative analysis with a large insulinoma cohort.

## Methods

### Subjects

**Figure 1:**
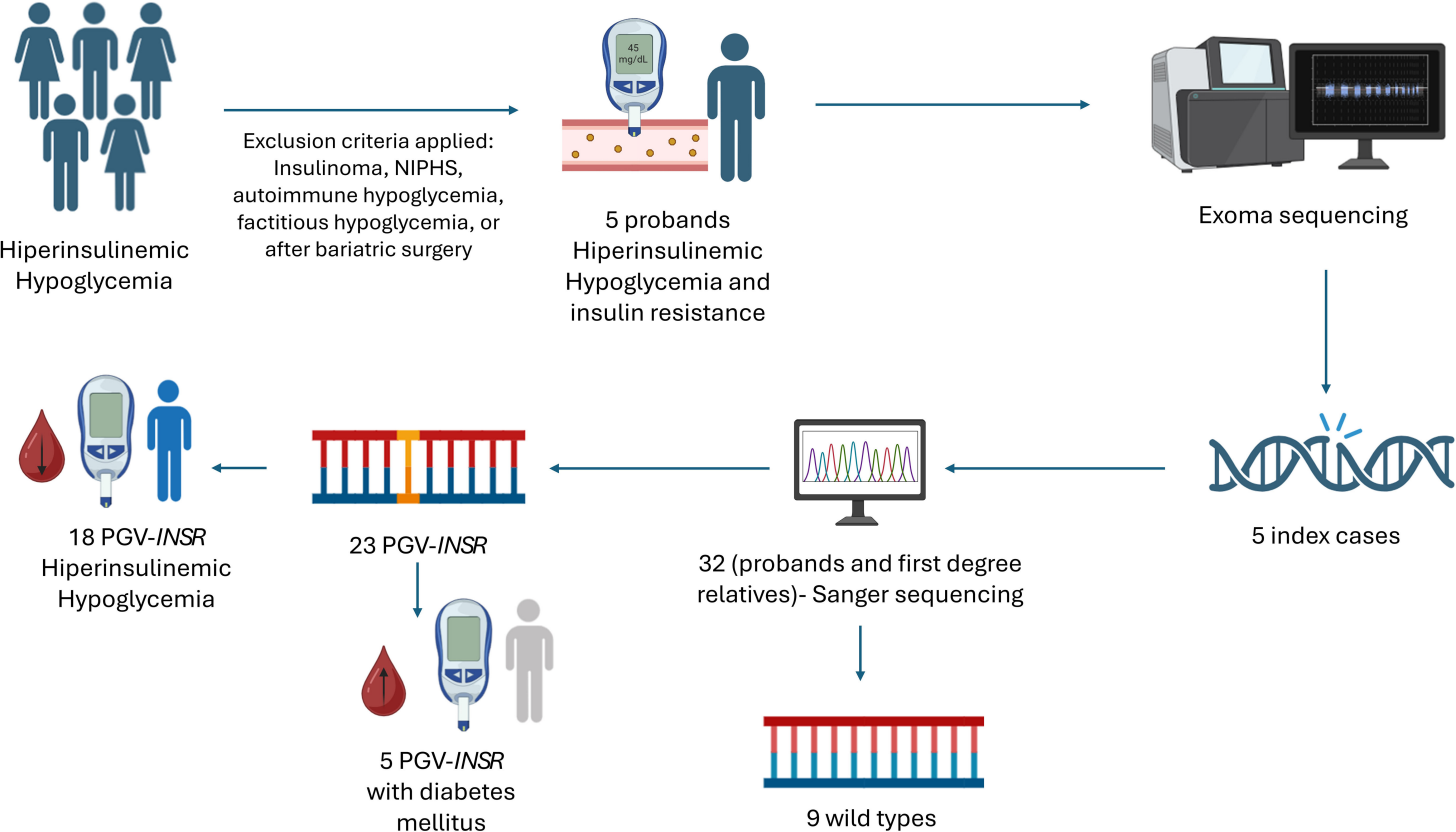
Flowchart illustrating patient selection and methodological workflow, including molecular evaluation of individuals with hyperinsulinemic hypoglycemia and IR who underwent exome sequencing and familial segregation by Sanger sequencing.

This retrospective cohort, single-center observational study was derived from a cohort of 94 individuals evaluated in a specialized hypoglycemia clinic between January 2020 and January 2025 at the Endocrinology Outpatient Service of Hospital das Clínicas, University of São Paulo (HCFMUSP). This tertiary academic center is a national and regional reference for complex endocrinopathies, receiving patients from across Brazil and Latin America through the public health system. We selected only normal-weight patients (BMI < 25 kg/m²) with clinical suspicion of hypoglycemia and signs of IR (acanthosis nigricans with or without PCOS). Patients with gastrointestinal surgery, autoimmune hypoglycemia, insulinoma or insulinomatosis, NIPHS, or factitious hypoglycemia were excluded (**Figure 1**).

### Metabolic analysis of hypoglycemic state and biochemical profile

Patients underwent a prolonged fasting test (PFT), a mixed-meal tolerance test (MMT), and a 75-g oral glucose tolerance test (75-OGTT). The PFT was conducted in accordance with the Clinical Practice Guidelines for the management of hypoglycemic disorders issued by the Endocrine Society (2). The test was terminated upon the development of hypoglycemic symptoms associated with capillary glucose ≤55 mg/dL, or, in the absence of symptoms, capillary glucose ≤45 mg/dL, or when capillary ketonemia exceeded 1.0 mmol/L. Peripheral venous blood samples were obtained for the measurement of plasma glucose, insulin, and C-peptide concentrations. Capillary β-hydroxybutyrate (ketonemia) was assessed using a point-of-care test strip (MediSense Optium).

Both the MMT and 75-OGTT were performed in the morning (07:00–08:00 h) after a 12-h overnight fast. The MMT consisted of a standardized mixed meal (~450 kcal; 63% carbohydrates, 28% fat, and 9% protein), with blood sampling for plasma glucose and insulin at baseline and every 30 min for 300 min; C-peptide was measured at baseline and at 300 min. The primary aim of the MMT was to assess postprandial hypoglycemia. The 75-OGTT involved oral administration of 75 g of glucose, with plasma glucose and insulin measured at identical time points to evaluate glucose tolerance and insulin secretory response, using the American Diabetes Association criteria for glycemic classification (24). For all metabolic tests, insulin, GLP-1 receptor agonists, and SGLT2 inhibitors were not in use, and metformin was discontinued at least 7 days prior to testing.

Plasma glucose, insulin, and C-peptide were measured at baseline and serially during each test. β-Hydroxybutyrate (ketonemia) was assessed in capillary blood (MediSense Optium). Hyperinsulinemic hypoglycemia was defined as glucose ≤55 mg/dL with symptoms or ≤45 mg/dL without symptoms, associated with insulin ≥3 μU/mL, C-peptide ≥0.6 ng/mL, β-hydroxybutyrate <1 mmol/L, and negative screening for oral hypoglycemic agents. The insulin/C-peptide molar ratio (In/PeptC) was calculated after 12 h of fasting.

Serum insulin concentrations were measured using a chemiluminescent microparticle immunoassay on the Alinity i analyzer (Abbott Diagnostics, Chicago, United States), with an analytical range of 1.6–300 µU/mL. As insulin concentrations in PGV-*INSR* samples frequently exceeded the upper limit of quantification, a standardized dilution protocol was applied, including automated 1:2 dilution and, when necessary, additional manual 1:10 dilutions using low-insulin serum. Final concentrations were calculated by applying the appropriate dilution factors.

Fasting triglycerides were measured in index cases after a 12-h fast using an enzymatic colorimetric method. Values <150 mg/dL were considered within the normal reference range.

### Whole-exome sequencing and bioinformatics analysis

Genomic DNA was extracted from peripheral leukocytes by the salting-out method. Whole-exome sequencing was performed on the Illumina platform (GRCh37 reference genome) and analyzed using a custom pipeline (v3.10). Variants were described according to the Human Genome Variation Society (HGVS) nomenclature and classified by the American College of Medical Genetics and Genomics/Association for Molecular Pathology (ACMG/AMP) guidelines, integrating population databases (gnomAD, ABraOM, 1000 Genomes, and TOPMed, among others), *in silico* predictions, segregation, and literature data (25).

### Familial segregation

Variants detected in probands were tested in at-risk relatives by Sanger sequencing. Exon-specific primers covering exons 18–21 were used, as previously described (26). PCR products were purified and sequenced on an ABI Prism 3130XL Genetic Analyzer. Segregation strength (PP1) was evaluated following Jarvik and Browning thresholds (27).

### Ethics

The study was approved by the Ethics Committee of the University of São Paulo Medical School (CAAE: 66121022.5.0000.0068). All participants provided written informed consent.

### Use of artificial intelligence tools

ChatGPT (OpenAI, San Francisco, USA) was used to assist with grammar review, English translation, statistical analysis support, and figure preparation. AI was not used for data generation or interpretation. All outputs were reviewed and validated by the authors.

### Statistical analysis

Clinical and demographic data are presented as medians with 25th and 75th percentiles. OGTT responses were compared by area under the curve (AUC) using the non-parametric Mann–Whitney test. Comparisons were performed between patients with PGV-*INSR*-related hypoglycemia and a previously reported insulinoma cohort (28). Statistical significance was defined as *p <*0.05.

### Literature review

The literature review was conducted by searching the PubMed, Cochrane Library, and LILACS databases using the Medical Subject Headings (MeSH) terms “INSR mutation” and “hyperinsulinemic hypoglycemia” as well as references cited in articles initially identified through these search terms. The search was limited to studies published between January 2000 and December 2025. Duplicate publications were excluded, and cases reported in more than one article were carefully identified and counted only once to avoid duplication of patients.

## Results

### Clinical features

**Figure 2:**
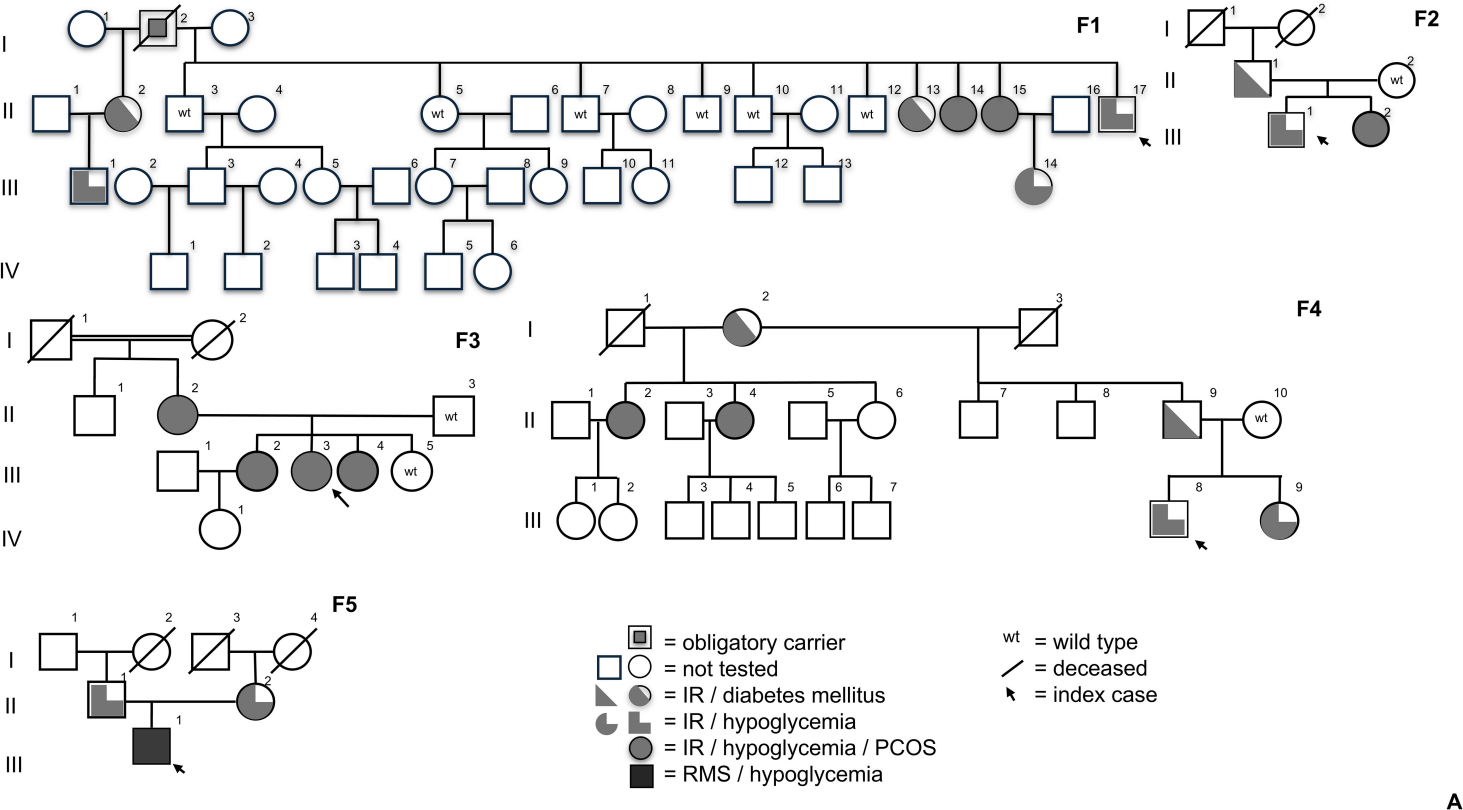
Pedigrees of the five families carrying PGV*-INSR*, including four families with heterozygous variants and one family with Rabson–Mendenhall syndrome (RMS) due to homozygosity. The figure illustrates the phenotypic variability observed, encompassing IR/diabetes mellitus, IR/hypoglycemia, IR/hypoglycemia/PCOS, and RMS/hypoglycemia. IR, Insulin resistance; PCOS, Polycystic Ovary Syndrome; RMS, Rabson Mendenhall Syndrome; PGV-INSR, Pathogenic germline variant – Insulin receptor gene.

IR, insulin resistance; PCOS, polycystic ovary syndrome; RMS, Rabson–Mendenhall syndrome; PGV-*INSR*, pathogenic germline variant-insulin receptor gene.

Five unrelated index cases were included: three were referred to our service due to hypoglycemia as the main initial clinical manifestation, one patient and family members with PCOS and TAIRS for investigation of hypoglycemia, and another due to RMS and diabetes preceded by a history of hypoglycemia. All exhibited marked IR and BMI <25 kg/m² (**Figure 2**). The median age at diagnosis of the five probands was 22 (20–25) years. Molecular investigation identified PGV-*INSR* in all five index cases through exome analysis (**Supplementary Figure 1**) and enabled genetic screening of at-risk relatives by Sanger sequencing. In total, 32 individuals were genetically investigated, including probands and first degree relatives, of whom 23 carried PGV-*INSR* with variable and clinically relevant degrees of IR, 18 were associated with hypoglycemia documented by at least one of the metabolic evaluation tests performed (FPG, OGTT, and/or MTT), while 5 had a prior diagnosis of diabetes at the time of the initial assessment, and 9 wild types (**Figure 2**). The detailed clinical history and course of each index case, as well as clinical features of PGV-*INSR*-positive relatives, are also provided (**Supplementary Material**).

### Molecular findings

**Figure 3:**
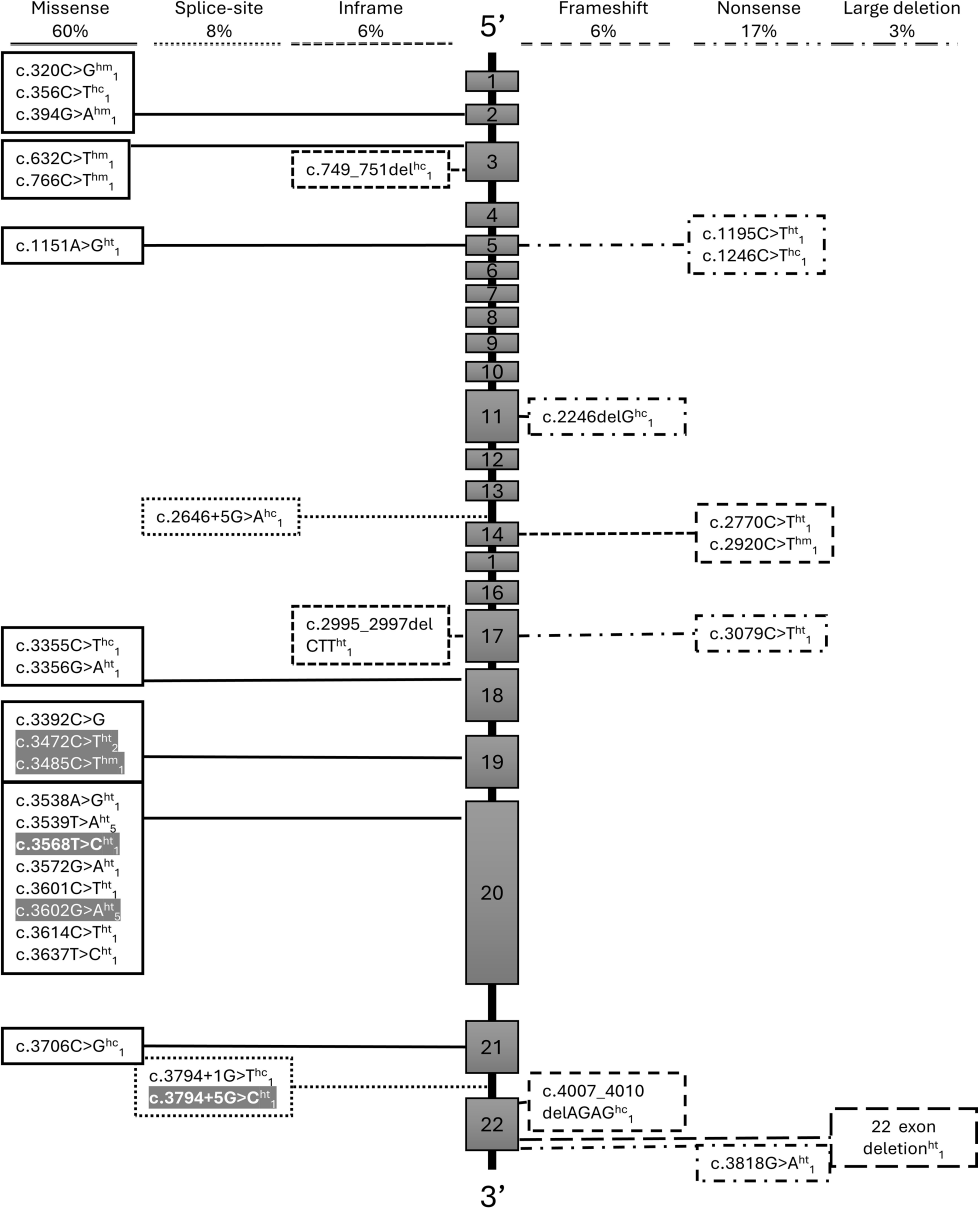
Schematic distribution of PGV-*INSR* across exons and introns of the *INSR* gene, highlighting recurrently affected regions. In gray, the variants identified in this study; in bold, the INSR pathogenic variants (PGV-INSR) with the first description associated with the hypoglycemia phenotype; subscript numbersindicate the count of probands reported in the literature. Missense, splice-site, and in-frame PGV-INSR are shown on the left, whereas frameshift, nonsense, and large deletions are shown on the right. INSR, insulin receptor gene; PGV, pathogenic germline variant; Ht, simple heterozygosity; Hm, homozygosity; Hc, compound heterozygosity. Gray boxes represent each of the 22 exons of the INSR gene.

**Figure 4:**
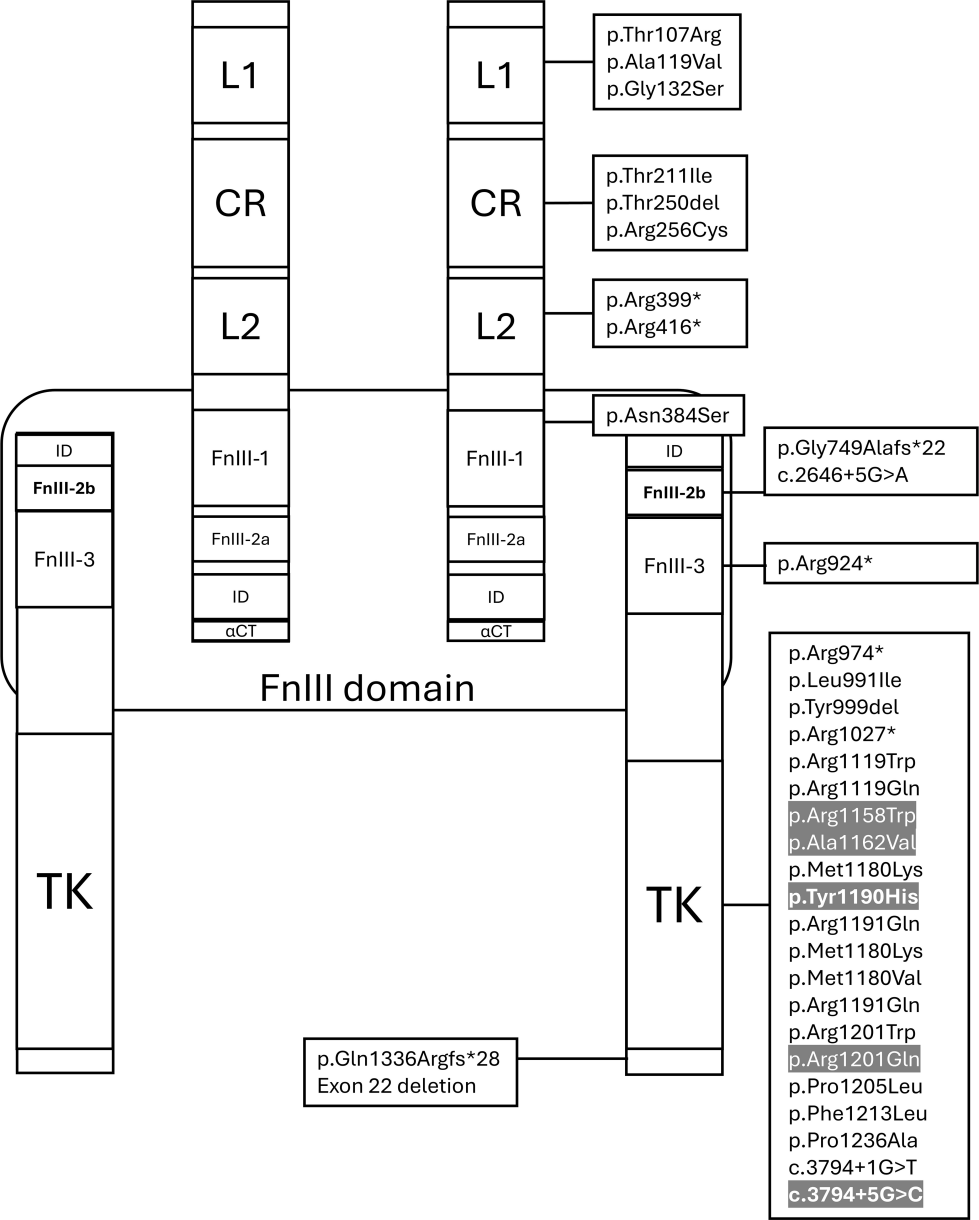
Functional domain mapping of PGV-*INSR*, showing predominance in the β-subunit and catalytic tyrosine kinase domain. In gray, the variants identified in this study; in bold, the INSR pathogenic variants (PGV-INSR) with the first description associated with the hypoglycemia phenotype. L1, leucine-rich domain 1; CR, cysteine-rich region; L2, leucine rich domain 2; FnIII1, fibronectin type III domain 1; FnIII2a, fibronectin type III domain 2a; TK, catalytic tyrosine kinase domain. The symbol (*) indicates a stop codon.

**Supplementary Table 1** (**Supplementary Material**) summarizes the PGV-*INSR* identified in the five probands, detailing zygosity, localization, type, classification (ACMG/AMP criteria), population frequencies, and previous reports. All variants were classified as pathogenic or likely pathogenic based on multiple lines of evidence (PS4, PM2, PM1, PP1, PP2, PP3, PM3, PS3). Four were missense variants, and one was a splice-site alteration. PGVs in families 1–4 were present in heterozygosis, while the variant in family 5 was in homozygosis: family 1, c.3602G>A (p.Arg1201Gln); family 2, c.3472C>T (p.Arg1158Trp); family 3, c.3568T>C (p.Tyr1190His); family 4, c.3794 + 5G>C; and family 5, c.3485C>T (p.Ala1162Val). All variants identified were located between exons 19 and 21, which are associated with the tyrosine kinase domain of the INSR, as illustrated in **Figures 3**, **4**, and were compiled as PGV-INSR in the present study, as all PGVs previously reported were associated with hypoglycemia (**Figures 3**, **4**).

### Glucose tolerance test profiles

**Figure 5:**
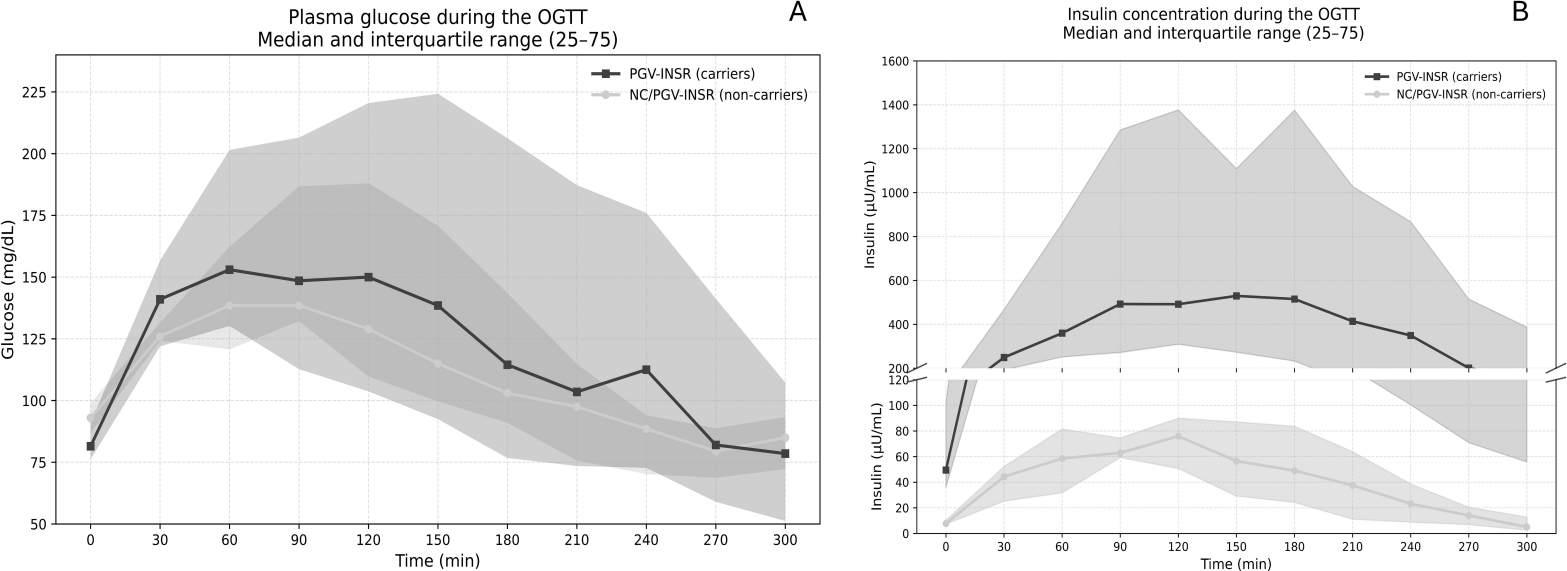
**(A)** Glucose profiles during the 75-g OGTT in PGV-*INSR* carriers (*n* = 22) versus non-carriers (*n* = 6). Values represent median (P25–P75). **(B)** Insulin profiles during the 75-g OGTT in PGV-*INSR* carriers (*n* = 22) versus non-carriers (*n* = 6). Values represent median (P25–P75); *p* < 0.05 AUC using the Mann–Whitney test. Despite severe insulin resistance, the PGV-INSR group exhibited a median fasting triglyceride concentration of 47 mg/dL after a 12-h fast, with an interquartile range (25th–75th percentiles) of 42–54 mg/dL.

A hormonal and biochemical response profile during the 75-g OGTT was provided by comparative analysis of 22 PGV-*INSR* carriers and 6 non-carrier family members. One additional carrier (III-9, family 4) was unable to complete the OGTT due to intolerance to dextrose, despite two attempts, and was therefore excluded from OGTT-based analyses. Among the nine asymptomatic wild-type relatives, three declined to undergo the OGTT and MMT, resulting in six non-carriers included in the comparative tests (**Supplementary Table 2**). Glucose responses were similar between groups, peaking at 60–90 min (**Figure 5A**). In contrast, carriers exhibited exaggerated hyperinsulinemia, with median concentrations higher than non-carriers (*p* < 0.05 at all time points) (**Figure 5B**). Clinical IR manifested as acanthosis nigricans in only 4 of 23 carriers (17.4%) (**Supplementary Figure 2**).

### Hypoglycemia patterns

**Table 1** details the timing of hypoglycemic episodes in the five index cases, revealing distinct profiles according to the test performed. In the PFT, individuals II-17/F1, III-8/F4, and III-1/F5 exhibited plasma glucose levels between 54 and 55 mg/dL, associated with elevated insulin concentrations (19.0, 29.7, and 220.6 µU/mL, respectively), confirming the lack of appropriate suppression of insulin secretion. During the MMT, individual III-3/F3 presented with severe hypoglycemia (26 mg/dL) accompanied by insulin levels of 304 µU/mL, and III-8/F4 showed glucose of 46 mg/dL with insulin of 227.9 µU/mL—both illustrating an unexpected hypoglycemia with postprandial hyperinsulinemic response. In the 75-OGTT, individual III-1/F2 reached a nadir glucose level of 45 mg/dL with insulin of 561.5 µU/mL, and III-3/F3 exhibited glucose of 33 mg/dL with insulin of 164 µU/mL; additionally, III-8/F4 experienced another hypoglycemic episode (44 mg/dL) with insulin of 128.7 µU/mL. These findings demonstrate that, although all cases presented hypoglycemia associated with inadequate insulin suppression, the timing of occurrence varied among fasting, postprandial, and oral glucose-stimulated conditions.

**Table 1:** Hypoglycemic episodes documented in probands with PGV-*INSR*, according to the provocative tests: prolonged fasting test (PFT), mixed-meal test (MMT), or oral glucose tolerance test (75-OGTT)*.

Index case	PFT		MMT		75-OGTT	
	Glucose nadir (mg/dL)	Insulin (µU/mL)	Glucose nadir (mg/dL)	Insulin (µU/mL)	Glucose nadir (mg/dL)	Insulin (µU/mL)
II-17/F1	**54**	**19**	81	205	85	328.4
III-1/F2	67	23.8	84	279	**45**	**561.5**
III-3/F3	59	5	**26**	**304**	**33**	**164**
III-8/F4	**55**	**29.7**	**46**	**227.9**	**44**	**128.7**
III-1/F5	**55**	**220.6**	Not available		Not available	

American Diabetes Association criteria (24). The corresponding glucose values ≤55 mg/dL and their respective insulin concentrations are highlighted in bold.

### Comparison with insulinoma

**Table 2** compares PGV-*INSR* probands (*n* = 5) with insulinoma patients (*n* = 103). Carriers were younger and leaner. They also had greater fasting tolerance, and glycemic nadirs were higher in PGV-*INSR* carriers. While insulin concentrations were comparable, serum C-peptide levels were lower, resulting in higher insulin-to-C-peptide ratios. Notably, ketonemia was significantly higher in PGV-*INSR* probands, contrasting with normal values in insulinoma cases.

**Table 2:** Clinical and biochemical comparison between probands with PGV-*INSR* (*n* = 5) and patients with insulinoma (*n* = 103).

Variables	Insulinoma group (28)	PGV-*INSR* index case	*P*-value
Sex, F (%)	54.4%	20%	
Age (years)	40 (27–52)	20 (20–25)	<0.0102*
BMI (kg/m²)	26 (23.22–32)	22.9 (21.2–23.6)	0.0207*
Time elapsed until diagnosis (years)	16 (6–19)	24 (12–48)	0.1175
Fasting time (h)	6 (3–10)	22 (12–46)	0.0028*
Nadir glucose (mg/dL)	31 (26–38)	55 (55–59)	0.002*
Insulin (µU/mL)	23.8 (18–29.7)	20.2 (11–34.7)	0.7361
C-peptide (ng/dL)	3.8 (2.4–5.8)	0.98 (0.9–1.33)	0.0139*
Ins/PepC	0.12 (0.08–0.16)	0.47 (0.4–0.5)	0.0013*
Ketonemia (mmol/L)	0.1 (0.1–0.2)	1.1 (1.1–1.3)	0.0009*

Data are expressed as median and interquartile range.

*p*-value, bilateral *p*-value; F, female; BMI, body mass index.

*Comparison of median: insulinoma group vs. PGV-*INSR* by the Mann–Whitney test.

### PGV-*INSR* distribution associated with HH plus literature review included

All PGV-*INSR* associated with HH reported to date, including those from the present study, were compiled through a literature review (**Table 3**; **Figures 3**, **4**). Among 34 distinct variants, most were missense (59%), followed by nonsense (17%), splice-site (9%), in-frame deletions (6%), frameshift variants (6%), and one large deletion (3%). Exon 20, encoding the tyrosine kinase domain, accounted for 23.5% of all variants (8/34). Overall, 71.4% were localized to the β-subunit, predominantly within the catalytic tyrosine kinase region (**Figure 4**).

**Table 3:** Distribution of PGV-*INSR* associated with hyperinsulinemic hypoglycemia, according to mutation type, genomic location, affected protein domain, and hypoglycemia phenotype.

Variant type	cDNA	Zygosity	HGV	Receptor subunit	Hypoglycemia type	Patients	Syndromic features	Reference
M	c.320C>G	Hm	p.Thr107Arg	α	J	1	+^DS^	Rojek A, 2023 (29)
M	c.356C>T	Hc	p.Ala119Val	α	J	1	+^DS^	Grasso V, 2013 (30)
M	c.394G>A	Hm	p.Gly132Ser	α	J	1	+^RMS^	Abdelaziz RB, 2016 (31)
M	c.632C>T	Hm	p.Thr211Ile	α	J	1	+^DS^	Perge K, 2020 (32)
IF	c.749_751del	Hc	p.Thr250del	α	J	1	+^DS^	Zhou Q, 2021 (33)
M	c.766C>T	Hm	p.Arg256Cys	α	PP+J	1	−	Kuroda Y, 2015 (34)
M	c.1151A>G	Ht	p.Asn384Ser	α	PP+J	1	−	Guzman H, 2024 (35)
N	c.1195C>T	Ht	p.Arg399*	α	J	1	−	Ardon O, 2014 (26)
N	c.1246C>T	Ht	p.Arg416*	α	PP/J	2	−	Poon SWY, 2025 (36)
FS	c.2246delG	Hc	p.Gly749Alafs*22	β	J	1	+^RMS^	Yu L, 2022 (37)
SS	c.2646+5G>A	Hc		β	J	#	+^RMS^	Yu L, 2022 (37)
N	c.2770C>T	Hm	p.Arg924*	β	NE	1	+^DS^	Azzabi O, 2016 (38)
N	c.2920C>T	Hm	p.Arg974*	β	NE	1	+^DS^	Grasso V, 2013 (30)
IF	c.2995_2997delCTT	Ht	p.Tyr999del	β	N	1	−	Enkhtuvshin B, 2015 (39)
N	c.3079C>T	Ht	p.Arg1027*	β	PP+J	1	−	Imamovic M, 2024 (40)
M	c.3355C>T	Hc	p.Arg1119Trp	β	J	#	+^DS^	Zhou Q, 2021 (33)
M	c.3356G>A	Ht	p.Arg1119Gln	β	N	1	−	Sethi A, 2020 (20)
M	c.3392C>G	Hc	p.Pro1131Arg	β	J	1	+^RMS^	Yan, 2025 (41)
M	c.3472C>T	Ht	p.Arg1158Trp	β	NE	2	−	Krishnamurthy, 2016 (42)
M	c.3472C>T	Ht	p.Arg1158Trp	β	PP	2	−	You W, 2022 (43)
M	c.3472C>T	Ht	p.Arg1158Trp	β	PP	2	−	Nascimento RM, 2025 (present study)
M	c.3485C>T	Hm	p.Ala1162Val	β	J	1	+^RMS^	Nascimento RM, 2025 (present study)
M	c.3485C>T	Ht	p.Ala1162Val	β	PP	2	−	Nascimento RM, 2025 (present study)
M	c.3538A>G	Ht	p.Met1180Val	β	PP	1	−	Preumont V, 2016 (44)
M	c.3539T>A	Ht	p.Met1180Lys	β	J	2	−	Sethi A, 2020 (20)
M	c.3539T>A	Ht	p.Met1180Lys	β	PP	1	−	Prehn EL, 2025 (45)
M	c.3539T>A	Ht	p.Met1180Lys	β	NE	1	−	Crowley MT, 2024 (46)
M	**c.3568T>C**	**Ht**	**p.Tyr1190His**	**β**	PP	4	−	**Nascimento RM, 2025 (present study)**
M	c.3572G>A	Ht	p.Arg1191Gln	β	N	1	−	Sethi A, 2020 (20)
M	c.3601C>T	Ht	p.Arg1201Trp	β	PP	2	−	Huang Z, 2009 (47)
M	c.3602G>A	Ht	p.Arg1201Gln	β	PP	10	−	Højlund K, 2004 (22)
M	c.3602G>A	Ht	p.Arg1201Gln	β	PP	1	−	Grasso V, 2013 (30)
M	c.3602G>A	Ht	p.Arg1201Gln	β	PP	1	−	Preumont V, 2016 (44)
M	c.3602G>A	Ht	p.Arg1201Gln	β	PP+J	5	−	Nascimento RM, 2025 (present study)
M	c.3614C>T	Ht	p.Pro1205Leu	β	PM	1	−	Wei C, 2016 (48)
M	c.3637T>C	Ht	p.Phe1213Leu	β	PP	3	−	Innaurato S, 2018 (49)
M	c.3706C>G	Ht	p.Pro1236Ala	β	PM	1	−	Wei C, 2016 (48)
SS	c.3794+1G>T	Hc		β	NE	#	+^DS^	Grasso V, 2013 (30)
SS	**c.3794+5G>C**	**Ht**		β	PP+J	4	−	**Nascimento RM, 2025 (present study)**
N	c.3818G>A	Ht	p.Trp1273*	β	PP	2	−	Kuroda Y, 2015 (34)
FS	c.4007_4010delAGAG	Hc	p.Gln1336Argfs*28	β	J	#	+^RMS^	Yan, 2025 (41)
LD	Deleção do exon 22	Ht		β	PP+J	1	−	Verdecchia F, 2020 (50)
Total	34					67		

NM_000208.2. In bold, PGV-*INSR* was first reported in association with the hypoglycemia phenotype. Variants recurrently reported are outlined with borders.

M, missense; NS, nonsense; FS, frameshift; IF, in-frame deletion or insertion; SS, splice site; LD, large deletion; Ht, simple heterozygous; Hc, compound heterozygous; Hm, homozygous; E, exon; I, intron; CR, cysteine-rich domain; L1, leucine-rich domain 1; L2, leucine-rich domain 2; FnIII1, fibronectin type III domain 1; FnIII2, fibronectin type III domain 2; TK, tyrosine kinase domain; F, fasting; PP, postprandial; NS, not specified; PM, post-metformin; N, neonatal; #, individual carrying an additional PGV-*INSR* in compound heterozygosity. RMS, Rabson–Mendenhall syndrome; DS, Donohue syndrome. In bold, PGV-INSR was first reported in association with the hypoglycemia phenotype

In bold, PGV-INSR was first reported in association with the hypoglycemia phenotype.

A total of 20 distinct PGV-*INSR* in simple heterozygosity were identified in 56 individuals with HHF5 or TAIRS, encompassing 14 sporadic cases and 8 previously reported families. Overall, six homozygous PGV-*INSR* were linked to syndromic forms, including case III-1/F5 (c.3485C>T; p.Ala1162Val) with RMS, and eight compound heterozygous PGV-*INSR* combinations were identified in four syndromic cases (**Table 3**). In one case harboring homozygous PGV-*INSR*, syndromic phenotypic features were not reported (34).

**Figure 6:**
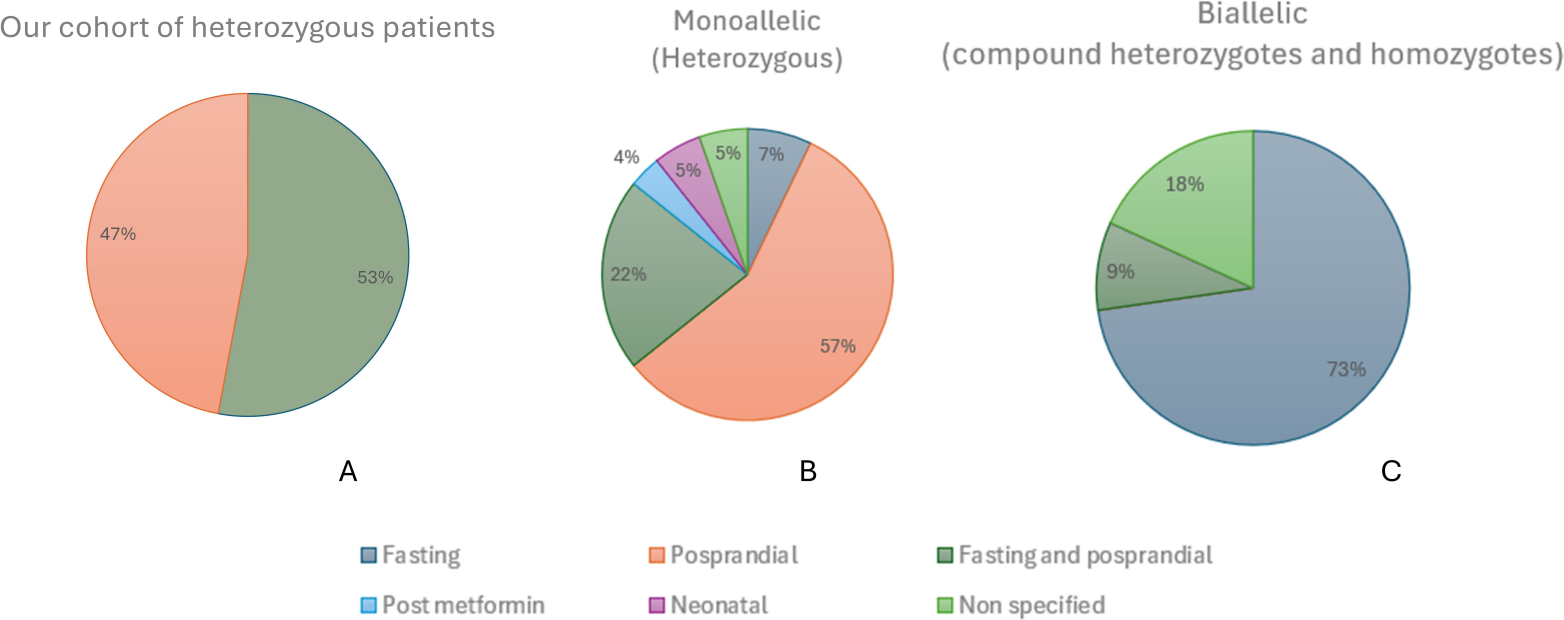
Distribution of the timing of hypoglycemia episodes among carriers of pathogenic *INSR* variants, including 18 cases of the present study and 49 from the literature. **(A)** Proportion of fasting, postprandial, and mixed hypoglycemia in our cohort of 17 heterozygous carriers. **(B)** Combined distribution including our 17 monoallelic heterozygous carriers and 39 heterozygous cases reported in the literature. **(C)** Distribution observed in our homozygous proband and in 10 additional biallelic *INSR* variant carriers previously reported.

In our cohort of 17 heterozygous PGV-*INSR* cases, the predominant pattern was combined postprandial and fasting hypoglycemia (53%), followed by postprandial hypoglycemia (47%) (**Table 3**; **Figure 6**). Considering all 56 heterozygous PGV-*INSR* patients reported to date—including those from the present study—postprandial hypoglycemia was likewise the most frequent presentation, occurring as an isolated form in 57% and combined with fasting hypoglycemia in 22%, whereas isolated fasting hypoglycemia was observed in 7% of cases (**Table 3**; **Figure 6**) followed by neonatal hypoglycemia (7%), unspecified (5%), and metformin-induced hypoglycemia (3%). Conversely, a different distribution was noted among the 11 patients with biallelic PGV-*INSR* forms, our case included, with predominance of fasting hypoglycemia (73%; 8/11) (**Table 3**; **Figure 6**).

### Semidominant inheritance

Considering the phenotype and the presence of heterozygous PGV-*INSR* carriers, three families (F1, F2, and F4) were clinically categorized as HHF5 by the presence of overt hypoglycemia in two or more family members. Family 3, due to the coexistence of PCOS and IR in four heterozygous women for PGV-*INSR*, was classified as TAIRS, while the proband of family 5, who presented with the classical RMS phenotype and harbored a homozygous PGV-*INSR*, was categorized accordingly. In these latter families, three members of the parental generation (F3 and F5) and two siblings (F3), all heterozygous PGV-*INSR* carriers, were diagnosed with IR and late postprandial hypoglycemia on testing, thereby supporting an additional classification of HHF5 in parallel with TAIRS and RMS, respectively. PCOS was diagnosed in other five women from families F1, F2, and F4, adding TAIRS to the HHF5 phenotype to these families. Thus, 60% (9/15) of the women had PCOS, sustaining an overlap of PGV-*INSR*-related phenotypes in these families.

## Discussion

### Principal findings

The present study describes the largest case series to date of individuals harboring PGV-*INSR* who manifested hypoglycemia. Our standardized laboratory protocol identified a distinctive metabolic signature during the PFT, characterized by hyperinsulinemic hypoglycemia with concomitant hyperketonemia, markedly elevated insulin concentrations at the time of hypoglycemia, increased insulin-to-C-peptide ratios during MMT, and normal serum triglyceride concentrations after 12 h of fasting.

In addition, we observed a substantial phenotypic overlap among *INSR*-related disorders—including HHF5, TAIRS, and RMS—across the five pedigrees analyzed. Notably, both dominant and recessive inheritance patterns coexisted within a single family, underscoring the clinical heterogeneity of PGV-*INSR* and highlighting important implications for diagnostic stratification, clinical management, and genetic counseling of affected individuals.

### Metabolic signature and pathophysiological insights

Compared with previously reported cohorts of insulinoma patients (28), PGV-*INSR* carriers in our study were younger and leaner, required prolonged fasting to induce hypoglycemia, and exhibited milder glycemic nadirs. Biochemically, they displayed lower C-peptide concentrations, markedly elevated insulin-to-C-peptide ratios, and—most notably—detectable ketonemia, a feature consistently absent in insulinoma (28).

This paradoxical profile reflects impaired insulin action at the hepatic level. Although insulin and C-peptide are secreted in equimolar amounts by pancreatic β cells, insulin undergoes rapid hepatic clearance, whereas C-peptide is predominantly eliminated by the kidneys. Pathogenic variants affecting the tyrosine kinase domain of the insulin receptor markedly reduce insulin metabolic clearance, leading to persistently elevated circulating insulin concentrations relative to C-peptide. This imbalance explains the increased insulin-to-C-peptide ratio and contributes to inappropriate hyperinsulinemia during hypoglycemia (51, 52).

The presence of ketonemia appears to represent a distinguishing feature compared with insulinoma and other forms of congenital hyperinsulinism, in which intact insulin receptor signaling typically suppresses hepatic ketogenesis. One possible explanation is that, in PGV-*INSR* carriers, systemic hypoglycemia leads to partial suppression of pancreatic insulin secretion into the portal circulation. The resulting reduction in hepatic insulin exposure, together with impaired receptor-mediated signaling, could allow ketone body production and limit hepatic glycogen storage despite elevated peripheral insulin concentrations. However, this mechanism remains hypothetical and requires further investigation.

The hypoketotic profile during fasting-induced hypoglycemia is well documented in gain-of-function PGV-*INSR* variants (35). In contrast, ketone dynamics in loss-of-function PGV-*INSR* remain poorly characterized (20, 36, 49, 50). Most available reports either measured β-hydroxybutyrate outside the hypoglycemic episode or described isolated cases with low venous concentrations during hypoglycemia, with occasional intrafamilial variability (20). Overall, the literature provides very limited data on ketone production during hypoglycemia in carriers of PGV-*INSR*, particularly during standardized fasting tests.

Oral glucose tolerance testing confirmed severe hyperinsulinemia at all time points, with fasting insulin concentrations frequently exceeding 25 µU/mL and post-stimulus peaks above 250 µU/mL. Despite this, classical clinical markers of insulin resistance, such as acanthosis nigricans, were present in only a minority of individuals, underscoring their limited sensitivity in receptor-level insulin resistance.

Consistent with receptor-level insulin resistance, fasting triglyceride concentrations were low, in contrast to the hypertriglyceridemia typically observed in post-receptor insulin resistance states, such as type 2 diabetes mellitus. This lipid profile further supports a distinct metabolic phenotype associated with PGV-*INSR*, resembling other insulin receptor disorders, including type B insulin resistance syndrome mediated by anti-insulin receptor antibodies (53).

Although an inclusion criterion of BMI <25 kg/m² was predefined as described in the *Methods* section (54), no individual with hypoglycemia fulfilling the biochemical criteria for hyperinsulinemic hypoglycemia was excluded due to excess body weight. Notably, all PGV-*INSR* carriers included in this study exhibited a BMI <25 kg/m². This finding is consistent with previous reports in cohorts of individuals harboring PGV-*INSR*, even in the absence of hypoglycemia, suggesting that a relatively low BMI may represent an intrinsic phenotypic feature of the disorder. From a pathophysiological perspective, this phenotype may reflect impaired adipose tissue expansion resulting from loss of insulin receptor function, leading to attenuation of insulin’s anabolic actions on adipocytes. Consequently, the capacity for lipid storage and weight gain may be intrinsically limited in these individuals. Supporting this concept, Musso et al. reported that among 11 patients with severe insulin resistance syndromes, including type A insulin resistance and Rabson–Mendenhall syndrome, all but one exhibited normal body weight (19). Collectively, these observations reinforce the association between insulin receptor dysfunction and the absence of significant adiposity, and they suggest that low or normal BMI may be a hallmark of the PGV-*INSR* phenotype, irrespective of the presence of hyperinsulinemic hypoglycemia.

### Genetic and structural considerations

All PGV-*INSR* identified in this study were located within the tyrosine kinase domain of the β-subunit of the *INSR*. Classically, hypoglycemia in heterozygous PGV-*INSR* carriers has been associated with variants affecting this catalytic domain (35). Indeed, among all 27 heterozygous PGV-*INSR* probands with hypoglycemia reported to date—including our four index cases—only three harbored variants in the α-subunit, outside the tyrosine kinase domain, reinforcing its pivotal role in the pathogenesis of HH (**Table 3**).

Notably, one of these exceptional cases involved an activating PGV-*INSR*, in contrast to all other *INSR*-mutated cases reported so far and, therefore, a distinctive entity of our present cases (35). The second case, with leprechaunism and hypoglycemia, had unexpectedly only monoallelic PGV-*INSR.* As this case was reported in 1992, it is possible that the second germline mutation, probably a large deletion, would be found with high-throughput sequencing based on NGS or by multiplex ligation-dependent probe amplification (26). The third case is the only one without syndromic features and may represent the first report of a single heterozygous pathogenic *INSR* variant located in the α-subunit of the insulin receptor. However, this patient underwent only exome sequencing (36). By exclusion of these cases, 80% (*n* = 16/20) of heterozygous PGV-*INSR* probands diagnosed with hypoglycemia had mutations within the tyrosine kinase domain, indicating that this region represents a mutational hotspot associated with hypoglycemia (**Figure 5**). These findings reinforce the pivotal—apparently exclusive—role of the tyrosine kinase domain in the pathophysiology of hypoglycemia in heterozygous cases. Thus, we recommended that hypoglycemia should be actively investigated in all carriers of PGV-*INSR* located in the tyrosine kinase domain of the insulin receptor.

Although our RMS case and hypoglycemia harbored a PGV-*INSR* located within the tyrosine kinase domain, syndromic cases with hypoglycemia revisited in the literature present homozygous or compound heterozygous variants dispersed in both the α- and β-subunits of the *INSR*, including the tyrosine kinase domain. Thus, this domain is not an exclusive region of the association of hypoglycemia and PGV-*INSR* heterozygous carriers (**Table 3**; **Figure 3**). In contrast, all variants outside the tyrosine kinase domain of the β-subunit or within the α-subunit were associated with syndromic forms (**Table 3**; **Figure 3**). Thus, we emphasize the importance of actively assessing hypoglycemia in syndromic cases, independently of the localization of the PGV-*INSR*. However, since a minority of cases with hypoglycemia have been reported carrying PGV-*INSR* outside the tyrosine kinase domain, it remains unclear whether obligatory heterozygous parents and siblings of syndromic patients (RMS or DS) with such variants might also develop hypoglycemia, as observed in the parental generation of our RMS proband carrying variants within the tyrosine kinase domain.

The c.3568T>C (p.Tyr1190His) variant, previously reported in family 3 by our group in association with PCOS (55), was shown to segregate with HH, thereby expanding its clinical spectrum. A novel splice-site variant, c.3794 + 5G>C, was identified and associated with HH, IR, and PCOS in family 4. The p.Arg1201Gln variant, present in four families—including family 1—is associated with the highest number of patients with HH (25%; 17/68) (**Table 3**) (22, 30, 44). The p.Arg1158Trp variant, found in proband 2, was previously associated with HH (42, 43), whereas the p.Ala1162Val variant, observed in homozygosity in proband 5, was previously reported in heterozygosity in association with PCOS (26, 56).

### Clinical implications of the superposition of *INSR*-related phenotypes

Clinical evaluation of the families included in this study revealed a combined expression of *INSR*-related phenotypes—HHF5, TAIRS, and RMS—with the remarkable coexistence of both dominant and recessive inheritance models within the family affected by RMS.

The presence of *INSR*-related HH is frequently associated with the HHF5 phenotype. Although families of the present study were initially categorized as HHF5 (F1, F2, and F4), TAIRS (F3), and RMS (F5), subsequent metabolic assessments identified additional relatives with hypoglycemia and IR in families F3 and F5, and individuals with PCOS, IR, or diabetes mellitus in families F1, F2, and F4, commonly linked to TAIRS, thereby demonstrating a clear superposition of *INSR*-related phenotypes.

The first and largest HHF5 family reported included 10 members with hypoglycemia and one with mild acanthosis nigricans and PCOS (22). Huang et al. described an adolescent girl with fasting hypoglycemia associated with hirsutism, hyperpigmented skin, acne, and oligomenorrhea, whose brother and father exhibited postprandial hypoglycemia and diabetes mellitus, respectively (47). In 2020, Sethi et al. reported a family with two siblings born small for gestational age presenting neonatal hypoglycemia, in addition to two unrelated sporadic cases with a similar phenotype (20).

Except for the Danish family, all HHF5 families reported to date are small, comprising up to two affected members with hypoglycemia, and are often described as isolated cases (**Table 3**). Even among these, in addition to hypoglycemia, clinical features such as acanthosis nigricans, primary or secondary amenorrhea, oligomenorrhea, hirsutism, and clitoromegaly have been documented, suggesting underlying PCOS and IR (20, 22).

These findings demonstrate that *INSR*-related disorders exhibit marked phenotypic variability both within and across families, encompassing a broad and overlapping clinical spectrum. As such, it may be inappropriate to classify families strictly according to individual *INSR*-related phenotypes—HHF5, TAIRS, or the syndromic forms (RMS and DS)—given the apparent and frequent superposition of these conditions among PGV-*INSR* carriers. Indeed, affected individuals may develop varying degrees of IR, hyperandrogenism, acanthosis nigricans, PCOS, diabetes, or even hypoglycemia. Collectively, these observations support the concept that the different *INSR*-related phenotypes represent components of a continuous clinical spectrum, emphasizing the need for active investigation of all these features in affected families.

Studies involving large families with genetic confirmation and long-term clinical follow-up may help determine the penetrance of each clinical feature and identify potential modifiers of the phenotype. In our cohort, the recognition of phenotypic superposition was made possible by the active genetic investigation of *INSR* carriers, which enabled the identification of mildly affected relatives and the delineation of the full phenotypic spectrum within families.

The broad clinical spectrum observed in *INSR*-related disorders suggests a semidominant inheritance pattern, in which disease severity depends on allele dosage (21). Syndromic forms such as RMS and DS, typically caused by biallelic *INSR* PGVs, present with severe manifestations consistent with recessive inheritance, whereas heterozygous carriers exhibit milder and more variable phenotypes compatible with autosomal dominant transmission. Recognizing the coexistence of these inheritance patterns within the same families is important for accurate genetic counseling, clinical management, and appropriate screening for hypoglycemia among first-degree relatives. Similar gene-dosage effects have been described in other disorders, including *CASR*-related disease (57–59)—where heterozygous variants cause familial hypocalciuric hypercalcemia and biallelic variants lead to severe neonatal hyperparathyroidism—as well as conditions associated with *SLCO2A1* (60–62) and *COL7A1* variants (63). Together, these examples illustrate how allele dosage can shape phenotypic variability across several human genetic diseases.

Very recently, Collin-Chavagnac et al. reported that members of the parental generation of five RMS probands exhibited clinical features of insulin resistance, including hyperandrogenism, hirsutism, PCOS, and diabetes, thereby linking TAIRS-like manifestations to these families (21). Based on these findings, the authors proposed a semidominant inheritance model in RMS. Our present data, together with the literature review, strengthen this hypothesis by documenting evidence for a continuous clinical spectrum of *INSR*-related disease ranging from mild to severe and syndromic forms, encompassing both classical Mendelian inheritance models within the same family.

PGV-*INSR* should be considered in the differential diagnosis of HH, particularly in young, lean patients presenting with inappropriate hyperinsulinemia, elevated insulin-to-C-peptide ratios, low triglyceride levels, and paradoxical ketonemia. Systematic genetic screening and counseling are warranted in affected families. These findings further expand the clinical spectrum of *INSR*-related disorders and underscore the value of integrating genetic testing in the evaluation of unexplained HH associated with IR.

### Study limitations

This study has some limitations that should be acknowledged. Its retrospective design may have limited the completeness and standardization of clinical and biochemical data. In addition, the cohort was assembled at a single tertiary referral center, which may affect the generalizability of the findings. Finally, although this represents the largest series of patients with hyperinsulinemic hypoglycemia associated with pathogenic variants in the *INSR* gene reported to date, the sample size remains limited, which is inherent to the rarity of the condition.

### Conclusions

This study reports the largest series of patients who harbor PGV-*INSR* presenting with HH and IR. Our findings reveal a continuum of *INSR*-associated phenotypes, including the semidominant inheritance pattern.

Our patients exhibited normal BMI and variable degrees of insulin resistance, with or without acanthosis nigricans, predominantly postprandial hypoglycemia, fasting and postprandial hyperinsulinemia, and normal fasting triglycerides. During PFT, a distinct metabolic pattern was observed, characterized by normoglycemia or hypoglycemia accompanied by hyperinsulinemia and ketonemia. This unusual combination—hyperinsulinemia in the presence of ketonemia—appears to represent a metabolic signature of PGV-*INSR*-related disorders. Recognition of this biochemical pattern may help clinicians suspect this rare condition and prompt targeted genetic investigation of the *INSR* gene.

In parallel, our data demonstrates a marked overlap among the classical INSR-related phenotypes—HHF5, TAIRS, and RMS—in the families reported herein. These findings expand both the phenotypic and molecular spectrum of *INSR*-related diseases and have implications for genetic counseling, emphasizing the potential occurrence of *INSR*-related manifestations in heterozygous relatives of RMS patients, as well as overlapping features between TAIRS and HHF5. Consequently, families harboring PGV-*INSR* should undergo comprehensive evaluation for the full range of clinical features associated with these apparently distinct entities.

## Data Availability

The original contributions presented in this study are included in the article and its Supplementary Material. Due to ethical, legal, and privacy restrictions related to patient confidentiality and the handling of human genetic data, individual-level datasets are not publicly available in open repositories. De-identified data supporting the findings of this study may be made available by the corresponding author upon reasonable request and with appropriate institutional and ethical approvals.
