# Supplementary material for: Hyperinsulinemic hypoglycemia due to pathogenic INSR variants: metabolic signature, phenotypic overlap, and semidominant inheritance
Source: Front Endocrinol (Lausanne). 2026 May 7;17:1716020. doi: 10.3389/fendo.2026.1716020 (PMC13191600; doi:10.3389/fendo.2026.1716020)
Supplement: Supplementary file 1 [file DataSheet1.pdf]

Supplementary material

Table S1. Molecular characterization of pathogenic and likely pathogenic *INSR* variants (PGV-*INSR*) identified in probands, including genomic position, zygosity, ACMG/AMP criteria applied, population frequency in genomic databases, and prior reports in the literature.

Index case	Variant	Variant Position	Zigozity	ACMG/AMP criteria	dbSNP	Prevalence	*In silico* predictor	References
**II-17/F1**	c.3602G>A: p.(Arg1201Gln)	E20	Ht	P (PS4,PP1,PM2,PM1,PP2,PM5,PP3,PS3)	rs121913156	0,0004% (gnomAD) Clinvar (14708)	D	Højlund et al. 2004(22); Grasso et al 2013(28); Preumont, et al 2017 (29) (HYPO)
**III-1/F2**	c.3472C>T: p.(Arg1158Trp)	E19	Ht	LP (PM2,PP2, PP3, PS4, PM5,PM1)	rs111993466	Absent	D	Krishnamurthy et al, 2016 (30); You et 2022(31); (HYPO)
**III-3/F3**	c.3568T>C: p.(Tyr1190His)	E20	Ht	LP (PP1, PM2, PM1, PP2, PP3)	rs1448499462	0,000657% (gnomAD)	D	Crespo et al, 2022(32) (PCOS)
**III-8/F4**	c.3794+5G>C	I21	Ht	LP (PM2, PP3, PP1)		Absent	D	present study
**III-1/F5**	c.3485C>T: p.(Ala1162Val)	E19	Hm	LP (PM2, PM3, PM5, PP2,PP3)	rs121913154	Clinvar (2637065)	D	Ardon, et al 2014(25); Cama et al 1992(33) (PCOS)

Abbreviations: Ht = heterozygous; Hm = homozygous; D = deleterious; E20 = exon 20; E19 = exon 19; I21 = intron 21. Variants according to NM_000208.4; NC_000019.9 (INSR_v002); P = Pathogenic; LP = Likely pathogenic. PCOS = previously reported with PCOS phenotype; HYPO (previously reported with hypoglycemia phenotype) ;dbSNP = Database of Single Nucleotide Polymorphisms; ACMG/AMP = American College of Medical Genetics and Genomics/ Association for Molecular Pathology: PS3 – Functional studies supportive of a damaging effect on the gene or gene product; ACMG/AMP criteria = PS4 – Increased prevalence of the variant in affected individuals and where missense variants are a common mechanism of disease; PP3 – Multiple computational predictions support a deleterious effect; PM1 – Located in a mutational hot spot or critical and well-established functional domain without benign variation; PM2 – Absent or extremely rare in population da; PM3 – Detected in trans with a pathogenic variant in a recessive disorder; PM5 – Novel missense change at an amino acid residue where a different pathogenic missense variant has been observed.

Table S2. Comparative glycemic and insulinemic responses during the 75 g OGTT in carriers of *INSR* pathogenic variants (PGV-*INSR*, n = 22) and non-carriers (n = 6). Data expressed as mean ± SD.

75g OGTT	Time in minutes		0	30	60	90	120	150	180	210	240	270	300
**Insulin (µU/mL)***	**PGV-*INSR***	Median (25-75)	49,45 (35.8 – 101.4)	248.95 (192.02 – 467.37)	359.75 (251.35 – 860.40)	492.45 (271.87 – 1285.61)	491.55 (309.31 - 1377)	529.45 (273.25-1109.47)	515.20 (232.6 – 1375.85)	413.85 (128,37-1027.90)	349.10 (100.37 – 867.52)	200.65 (70.85 – 514.86)	151.57 (55.75 – 387.18)
**Glucose (mg/dL)**		Median (25-75)	81.5 (76.25-91)	141 (122-156.75)	153 (130.25-201.5)	148.5 (112.75 – 206.5)	150 (103.75-220.5)	138.5 (92.5 – 224.25)	114.5 (76.75 – 206.25)	103.5 (73.5 – 187.25)	112.5 (72.75 - 176)	82 (59-141.25)	78.5 (51.25 – 107.25)
**Insulin (µU/mL)***	**NC/PGV-*INSR***	Median (25-75)	7.75 (7.27 – 10.1)	44.25 (25.25 – 52.22)	58.5 (31.72 – 81.52)	62.85 (59.3 – 74.5)	75.9 (50.72 – 89.97)	56.5 (29.22 – 87.03)	49 (24.25-83.65)	37.5 (11.15-63.7)	23.15 (8.85 – 38.57)	14 (6.87-20.37)	5 (2.62-12.77)
**Glucose (mg/dL)**		Median (25-75)	93 (87-99)	126 (124.25-131.5)	138.5 (120.75-162.25)	138.5 (132.25-186.75)	129 (109.75-188)	115 (99.75-170.75)	103 (91-143.5)	97.5 (75.75-114.75)	88.5 (70.25-94)	79.5 (68.75-88.75)	85 (72.25 – 93.25)
**AUC Insulin p-value***			0.0003*										
**AUC Glucose p-value**			0.6143										

75g OGTT = oral glucose tolerance test; PGV-*INSR* = pathogenic germline variant in *INSR*; NC, non-carrier; p < 0.05 = statistically significant; * = comparison of area under the curve between both groups (PGV-*INSR* vs. NC/PGV-*INSR*) using Mann-whitney test.

Family 1

(Proband II-17/F1) — A 29-year-old male reported recurrent episodes of weakness and malaise since adolescence, typically triggered by prolonged fasting or physical activity, and occasional late postprandial somnolence and hunger. He noted a mild weight gain (~5 kg) over recent years. Physical examination showed a BMI of 24.8 kg/m² and mild cervical acanthosis nigricans. Family history revealed an asymptomatic mother and a diabetic father who died prematurely; three sisters and one niece reportedly exhibited similar symptoms (**Figure 2**).

During a 12-hour fasting test (**Table S3**), glucose was 85 mg/dL, insulin 328 µU/mL, C-peptide 5.9 ng/mL, insulin-to-C-peptide ratio 1.2, and triglycerides 69 mg/dL, consistent with marked insulin resistance (IR). After 72 hours of fasting, hypoglycemia (54 mg/dL) occurred with concomitant hyperinsulinemia (18 µU/mL) and moderate ketonemia (1.9 mmol/L); the Ins/C-peptide ratio decreased to 0.4 but remained elevated.

Table S3. FPT of the index case II-17/F1 (Family 1).

Fasting time (hours)	Glucose (mg/dL)	Insulin (µU/mL)	C-peptide (ng/mL)	Ketonemia (mmol/L)	Ins/C-peptide
**12h**	85	328	5,9	0	1,2
**72h**	54	18	0,9	1,9	0,4

FPT = Fasting prolonged test

The OGTT (**Table S4**) confirmed diabetes at 120 minutes (226 mg/dL) despite normal fasting glucose, with basal and stimulated insulin levels markedly increased (328.4 and 2051.9 µU/mL, respectively).

Table S4. OGTT of the index case II-17/F1 (Family 1).

Time (minutes)	Glucose (mg/dL)	Insulin (µU/mL)
**0**	85	328,4
**30**	140	427,4
**60**	230	712,2
**90**	238	1124,4
**120**	226	1583,6
**150**	229	1696
**180**	219	2051,9
**210**	192	1972,5
**240**	173	1858,2
**270**	148	1761,6
**300**	136	1686,3

OGTT = Oral glucose tolerance test

The mixed-meal test (**Table S5**) did not induce hypoglycemia but showed persistently high insulin concentrations (basal 205 µU/mL; peak 834 µU/mL) and elevated Ins/C-peptide ratios (0.8–1.1), reinforcing a severe IR phenotype.

Table S5. MMT of the index case II-17/F1 (Family 1).

Time (minutes)	Glucose (mg/dL)	Insulin (µU/mL)	C-peptide (ng/dL)	Ins/C-peptide
**0**	81	205	5,6	0,8
**30**	104	284		
**60**	121	523		
**90**	105	590		
**120**	117	793		
**150**	117	805		
**180**	109	834		
**210**	100	600		
**240**	92	346		
**270**	94	633		
**300**	105	666	12,5	1,1

MMT = Mixed meal test

Family 2

Proband (III-1/F2): A 25-year-old male with recurrent episodes of sweating, malaise, tremor, and postprandial or fasting somnolence since the age of 4. At 12 years old, he experienced a loss of consciousness after a 12-hour fast while awaiting laboratory tests (maternal report). Physical examination showed weight 60.2 kg, height 162 cm, BMI 22.9 kg/m², and mild acanthosis nigricans on the neck and periorbital region (**Figure S2**). Family history included a sister (III-2/F2) with similar cutaneous findings, and a father diagnosed with diabetes mellitus; the mother and both sets of grandparents had no history of diabetes or similar symptoms.

Figure S2. Periorbital and cervical acanthosis nigricans (Proband: III-1/F2).

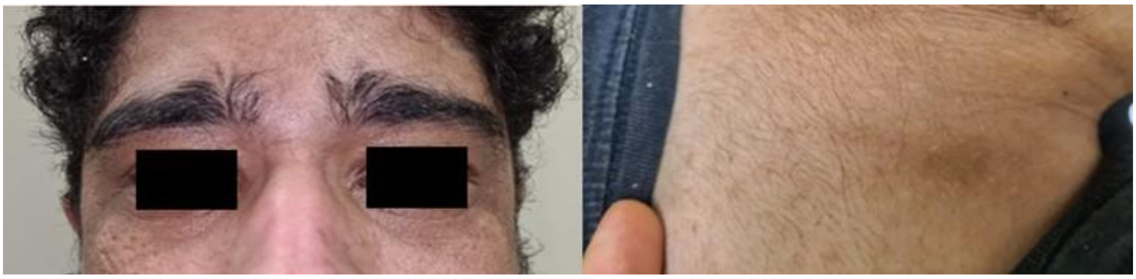

During a 3-hour fasting test, glucose was 81 mg/dL, insulin 126.7 µU/mL (771.6 pmol/L), C-peptide 3.04 ng/mL (845.1 pmol/L), insulin-to-C-peptide ratio 0.9, and triglycerides 47 mg/dL, consistent with marked insulin resistance. After 12 hours of fasting, glucose was 67 mg/dL, insulin 23.8 µU/mL, C-peptide 0.98 ng/mL (272.4 pmol/L), ketonemia 1.1

mmol/L (mild), and Ins/C-peptide ratio 0.5. The ratio decreased but remained elevated; the test was interrupted due to rising ketone levels (**Table S6**).

Table S6. FPT of the index case III-1/F2 (Family 2).

Fasting time (hours)	Glucose (mg/dL)	Insulin (µU/mL)	C-peptide (ng/mL)	Ketonemia (mmol/L)	Ins/ C-peptide
**3**	81	126,7 (771,6)	3,04 (845,12)	0	0,9
**12**	67	23,8 (144.9)	0,98 (272.4)	1,1	0,5

FPT = Fasting prolonged test

The OGTT revealed fasting glucose of 61 mg/dL with insulin 232 µU/mL. At 120 minutes, insulin peaked at 2471.3 µU/mL with paired glucose of 151 mg/dL, and at 300 minutes, a nadir glucose of 45 mg/dL with insulin 561 µU/mL was documented, confirming significant insulin resistance associated with hyperinsulinemic hypoglycemia (**Table S7**).

Table S7. OGTT of the index-case III-1/F2 (Family 2).

Time (minutes)	Glucose (mg/dL)	Insulin (µU/mL)	C-peptide (ng/mL)	Ins/C-peptide
**0**	61	232	3,16 (878,48)	1,6
**30**	122	1351,5		
**60**	131	1911,4		
**90**	149	2191,35		
**120**	151	2471,3		
**150**	137	2431,2		
**180**	111	1945,8		
**210**	110	1909,0		
**240**	78	1248,6		
**270**	49	851,0		
**300**	45	561,5		

OGTT = Oral glucose tolerance test

The MMT did not induce hypoglycemia but showed persistently high insulin levels (basal 279 µU/mL; peak >2429 µU/mL). The Ins/C-peptide ratio was elevated after a 12-hour fast (1.13) and increased further at the end of the test (1.86), reinforcing the presence of marked insulin resistance (**Table S8**).

Table S8. MMT of the index-case III-1/F2 (Family 2).

Time (minutes)	Glucose (mg/dL)	Insulin (µU/mL)	C-peptide (ng/mL)	Ins/C-peptide
**0**	84	279	5,39	1,13
**30**	123	470		
**60**	136	1540		

**90**	140	1404		
**120**	150	1091		
**150**	140	2141		
**180**	144	2038		
**210**	148	2233		
**240**	150	2429		
**270**	135	2254		
**300**	128	2079,2	24,36	1,86

MMT = Mixed meal test

Family 3

Proband (III-3/F3): A 20-year-old female who, since the age of 17, had complained of nonspecific malaise after 8 hours of fasting, along with acne, menstrual irregularity, and hirsutism on the face and chest. Physical examination showed weight 63.4 kg, height 164 cm, BMI 23.6 kg/m², clitoromegaly, mild cervical and marked axillary acanthosis nigricans. Family history included her mother (II-2/F3) with similar fasting-related malaise and a sister (III-2/F3) with two episodes of fasting-induced presyncope/syncope.

During a 12-hour fasting test, glucose was 80 mg/dL, insulin 24 µU/mL, C-peptide 1.96 ng/mL, Ins/PepC ratio 0.26, and triglycerides 54 mg/dL. Although fasting insulin was within the upper normal limit, clinical features were consistent with insulin resistance secondary to PCOS. Because of suspected hypoglycemia, a prolonged fasting test was performed. After 46 hours, glucose was 59 mg/dL, insulin 5 µU/mL, C-peptide 0.65 ng/mL, ketonemia 1.1 mmol/L (mild), and Ins/PepC ratio 0.16, which remained >0.1. The test was interrupted due to ketonemia (**Table S7**).

Table S9. FTP of the case-index III-3/F3 (Family 3).

Fasting time (hours)	Glucose (mg/dL)	Insulin (µU/mL)	C-peptide (ng/mL)	Ketonemia (mmol/L)	Ins/ C-peptide
**12h**	80	24 (146,16)	1,96 (544,88)		0,26
**46h**	59	5 (34,7)	0,65 (215,2)	1,1	0,16

FPT = Fasting prolonged test

The OGTT revealed normal glucose levels both at fasting (84 mg/dL) and at 120 minutes (82 mg/dL). However, basal (35.7 µU/mL) and peak insulin concentrations (1519 µU/mL) were markedly elevated relative to normal glucose values, indicating significant insulin resistance. Postprandial hypoglycemia occurred at 210 minutes, with a nadir glucose of 33 mg/dL and insulin of 164 µU/mL (**Table S10**).

Table S10. OGTT of the index-case III-3/F3 (Family 3).

Time (minutes)	Glucose (mg/dL)	Insulin (µU/mL)	C-peptide ng/dL
**0**	84	35,7	1,26

**30**	107	480,7	
**60**	111	909,8	
**90**	83	1357	
**120**	82	1519	
**150**	80	569	
**180**	26	304	
**210**	33	164	
**240**	92	103	
**270**	61	20	
**300**	65	12	0,3

OGTT = Oral glucose tolerance test

The MMT induced hypoglycemia at 180 minutes (glucose 26 mg/dL, insulin 304 µU/mL). Basal insulin was borderline (35.7 µU/mL), but the stimulated peak was markedly elevated (977 µU/mL at 120 minutes, with paired glucose of 82 mg/dL). The Ins/PepC ratio was high at baseline (0.62) and increased further by the end of the test (0.87). Elevated insulin concentrations in the presence of normal glucose levels and an increased Ins/PepC ratio confirmed a state of marked insulin resistance (**Table S11**).

Table S11. MMT of the index case III-3/F3 (Family 3).

Time (minutes)	Glucose (mg/dL)	Insulin (µU/mL)	C-peptide (ng/mL)	Ins/C-peptide
**0**	78	23	2,1	0,23
**30**	133	332		
**60**	116	446		
**90**	100	291,3		
**120**	107	452		
**150**	68	192		
**180**	53	66,8		
**210**	67	61		
**240**	71	42		
**270**	66	28		
**300**	74	19	1,48	0,28

MMT = Mixed meal test

Additional laboratory evaluation showed triglycerides 67 mg/dL, HbA1c 5.3%, total testosterone 306 ng/dL, free testosterone 196 pmol/L, and SHBG 37 nmol/L.

Family 4

Proband (III-8/F4): A 16-year-old male who, since the age of 10, had experienced fasting-induced dizziness and transient visual darkening, associated with marked acanthosis nigricans (Figure 2). His father (II-1) had been diagnosed with diabetes mellitus at age 27. Physical examination showed weight 75 kg, height 188 cm, BMI 21.2

kg/m², and pronounced cervical, axillary, and abdominal acanthosis nigricans (**Figure S3**).

Figure S3. Grade 4 acanthosis nigricans in the cervical and axillary regions of the proband from Family 4 (III-8/F4).

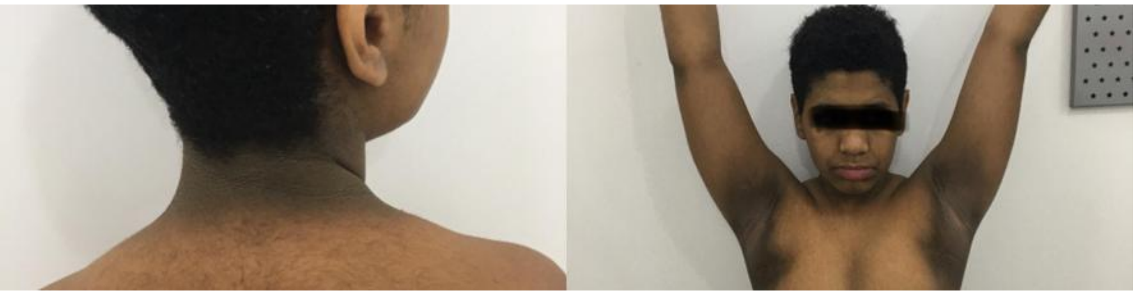

After a 16-hour fast, laboratory evaluation showed glucose 56 mg/dL, insulin 45.2 µU/mL, C-peptide 1.83 ng/mL, Ins/PepC ratio 0.54, ketonemia 0.5 mmol/L, and fasting triglycerides 42 mg/dL (12-hour fast). After 22 hours, hypoglycemia (55 mg/dL) was observed with insulin 29.7 µU/mL, C-peptide 1.33 ng/mL, Ins/PepC ratio 0.47 (VR < 0.1), and ketonemia 1.1 mmol/L. The prolonged fasting test confirmed hyperinsulinemic hypoglycemia with ketonemia and elevated Ins/PepC ratio, consistent with marked insulin resistance (**Table S12**).

Table S12. TPT of the index-case III-8/F4 (Family 4).

Fasting time (hours)	Glucose (mg/dL)	Insulin (µU/mL)	C-peptide (ng/mL)	Ketonemia (mmol/L)	Ins/ C-peptide
**16**	56	45,2	1,83	0,5	0,54
**22**	55	29,7	1,33	1,1	0,48

FPT = Fasting prolonged test

The OGTT showed normal fasting glucose (78 mg/dL) with elevated insulin (70.1 µU/mL). Insulin peaked at 725.1 µU/mL with paired glucose 149 mg/dL (120 min). Late postprandial hypoglycemia occurred at 210–300 minutes, with a nadir glucose of 44 mg/dL (240 min) and insulin 128.7 µU/mL. High basal and stimulated insulin levels with normal glucose and an elevated Ins/PepC ratio confirmed significant insulin resistance (**Table S13**).

Table S13. OGTT of the index-case III-8/F4 (Family 4).

Time (minutes)	Glucose (mg/dL)	Insulin (µU/mL)	C-peptide (ng/mL)	Ins/C-peptide
**0**	78	70,1	2,61	0,58
**30**	129	259,1		
**60**	146	425,7		
**90**	153	639,3		
**120**	149	725,1		
**150**	104	535,3		
**180**	74	179.6		

**210**	45	147.4		
**240**	44	128.7		
**270**	46	130.8		
**300**	49	84.7	2,16	0,85

OGTT = Oral glucose tolerance test

The MMT revealed normal fasting glucose (78 mg/dL) and elevated insulin (113.7 µU/mL), with a peak of 999.9 µU/mL at 120 minutes (glucose 122 mg/dL). Hypoglycemia occurred at 240–300 minutes, with a nadir glucose of 46 mg/dL (270 min) and insulin 227.9 µU/mL (**Table S14**). Persistently high basal and stimulated insulin concentrations relative to glucose, and an increased Ins/PepC ratio, demonstrated severe insulin resistance. Additional laboratory evaluation showed triglycerides 58 mg/dL and HbA1c 7.0%.

Table S14. MMT of the index-case III-8/F4 (Family 4).

Time (minutes)	Glucose (mg/dL)	Insulin (µU/mL)	C-peptide (ng/mL)	Ins/C-peptide
**0**	78	113,7	3,64	0,68
**30**	130	451,7		
**60**	154	796,8		
**90**	142	941,6		
**120**	122	999,9		
**150**	112	929,1		
**180**	88	755,1		
**210**	59	503,7		
**240**	47	358,8		
**270**	46	227,9		
**300**	50	163,3	2,62	1,29

MMT = Mixed meal test

Family 5

Proband (III-1/F5): A 20-year-old male was diagnosed at 11 months of age with asymptomatic fasting hypoglycemia and acanthosis nigricans. Family history included a father (II-1/F5) with systemic lupus erythematosus. Although the parents denied consanguinity, both share the same surname and were born in a small town of approximately 15,000 inhabitants in the countryside of Bahia State, Brazil.

The OGTT, performed at age 6 prior to the diagnosis of diabetes (**Table S15**), showed normal fasting glucose (61 mg/dL) and insulin 101.4 µU/mL, with a peak insulin of 2287.2 µU/mL and paired glucose of 121 mg/dL (120 minutes). The markedly elevated basal and stimulated insulin levels and increased Ins/PepC ratio (0.43 → 1.10) confirmed significant insulin resistance.

In 2016 (at age 13), during a symptomatic hypoglycemic episode after a 12-hour fast, laboratory results showed glucose 55 mg/dL, insulin 220.6 µU/mL, C-peptide 5.5 ng/mL,

HbA1c 7.1%, and Ins/PepC ratio 8.72 (**Table S16**). The elevated insulin, C-peptide, and Ins/PepC ratio values in the presence of low glucose and high HbA1c indicated marked insulin resistance. Fasting triglycerides were 26 mg/dL after a 12-hour fast.

Table S15. OGTT of the index case III-1/F5 (Family 5).

Time (minutes)	Glucose (mg/dL)	Insulin (µU/mL)	C-peptide (ng/mL)	Ins/C-peptide
**0**	61	101,4	5,1	0,43
**30**	138	679,7		
**60**	107	1240		
**90**	110	1725,60		
**120**	121	1778,90		
**150**	147	2287,20		
**180**	136	1601,04		
**210**	123	1072		
**240**	110	868,15		
**270**	117	514,89		
**300**	99	360,42	7,15	1,10

OGTT = Oral glucose tolerance test

Supplementary Table 14 summarizes disease progression and treatment history. Initially presenting with asymptomatic hypoglycemia, the patient developed diabetes mellitus at age 13 and started metformin therapy. Regular insulin was introduced at age 16, and he currently uses an NPH and regular insulin basal-bolus regimen at 2 IU/kg/day. Current physical examination: weight 50 kg, height 163 cm, BMI 18.8 kg/m², and mild cervical and axillary acanthosis nigricans.

Table S16. Longitudinal clinical follow-up of the proband from Family 5 (III-1/F5).

Age (years)	6 years	13 years	16 years	20 years
**Treatment**		Metformin 500mg	Metformin 1g + Regular insulin	NPH and Regular insulin
**HbA1c (%)**	5.8	7.1	9.6	11.4
**Fasting glucose (mg/dL)**	67	56	59	135
**Triglycerides (mg/dL)**	26			38
**C-peptide (ng/mL)**	4,3	5.5	6.97	2.95
**Insulin (µU/mL)**	168.6	220.6		

Figure S4. IGV alignment of exome sequencing reads showing the nucleotide substitutions identified in the five families carrying PGV-*INSR*

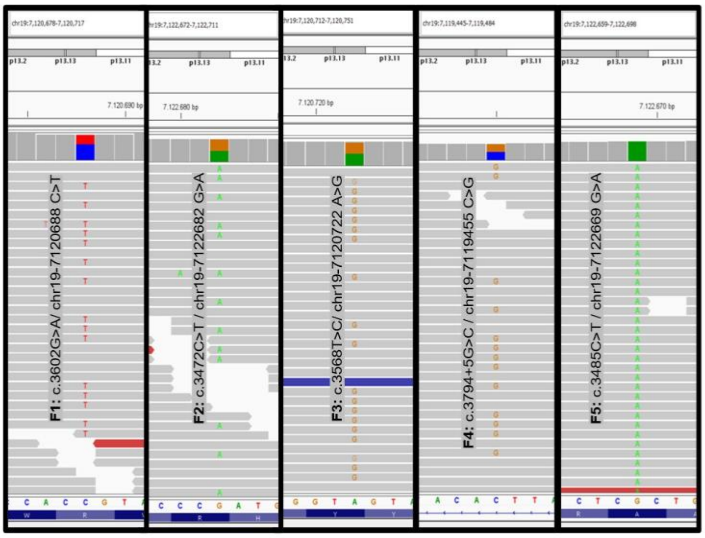
F1: c.3602G>A/ chr19-7120688 C>T
F2: c.3472C>T / chr19-7122682 G>A
F3: c.3568T>C/ chr19-7120722 A>G
F4: c.3794+5G>C / chr19-7119455 C>G
F5: c.3485C>T / chr19-7122669 G>A
